# Comparison of the Effectiveness of Ultrasound-Guided Proximal, Mid, or Distal Adductor Canal Block after Knee Arthroscopy

**DOI:** 10.5152/TJAR.2023.22225

**Published:** 2023-04-01

**Authors:** Aylin Tamam, Selin Güven Köse, Halil Cihan Köse, Ömer Taylan Akkaya

**Affiliations:** 1 Clinic of Anaesthesiology and Reanimation, Mamak State Hospital, Ankara, Turkey; 2 Clinic of Anaesthesiology and Pain Medicine, Kocaeli City Hospital, Kocaeli, Turkey; 3 Department of Anaesthesiology and Pain Medicine, Health Sciences University Dışkapı Yıldırım Beyazıt Training and Research Hospital, Ankara, Turkey

**Keywords:** Adductor canal block, knee arthroscopy, tramadol consumption

## Abstract

**Objective:**

Adductor canal block has been used for effective post-operative analgesia; however, the optimal location of adductor canal block placement is still controversial. We aimed to assess the opioid consumption and pain intensity in patients undergoing proximal, mid, and distal adductor canal block after knee arthroscopy.

**Methods:**

A total of 90 patients who had undergone an arthroscopic knee surgery and proximal, mid, or distal adductor canal block for post-operative analgesia were examined. All groups received 20 mL of bupivacaine (0.375%) to the adductor canal. Post-operative pain scores, tramadol consumption, Bromage scores, additional analgesic need, and other complications were recorded.

**Results:**

Our results demonstrated that proximal adductor canal block group significantly reduced opioid consumption compared to the mid-adductor canal block group (*P* < .001), and mid-adductor canal block group provided significantly decreased opioid consumption than the distal adductor canal block group (*P* = .004). The visual analog scale values were significantly lower in the proximal adductor canal block group compared to the mid-adductor canal block group at 0, 2, 4, 8, 12, and 24 hours, except in resting visual analog scale values at 24 hours. When the proximal and distal groups were compared, visual analog scale values were significantly lower in the proximal adductor canal block group. The Bromage score was 0 in all groups at each follow-up point. Post-operative nausea was observed in only 3 (3.3%) patients, all of these patients were in the distal adductor canal block group.

**Conclusion:**

Ultrasound-guided adductor canal block can be applied reliably at proximal, mid, and distal locations. The proximal adductor canal block approach provides significantly lower tramadol consumption and post-operative visual analog scale values than the mid- and distal adductor canal block groups.

**Main Points**

The ultrasound-guided techniques have gained popularity and have been adopted in regional anaesthesia and interventional pain medicine as an essential element of multimodal analgesia regimen.
Adductor canal block has been used for effective postoperative analgesia, without motor block.
Proximal adductor canal block is more advantageous regarding pain relief and opioid consumption compared to mid and distal blocks.

## Introduction

Arthroscopic knee surgery is a commonly performed orthopaedic procedure due to being minimally invasive and providing early recovery.^1^ Although using small incisions, a significant number of patients have severe pain in postoperative period that may lead to poor quality of recovery after operation.^2,3^ Currently, multimodal analgesia is a widely used method of pain relief, opioid burden, and length of patient’s stay in the hospital and also to improve patient functionality and satisfaction scores.^4^ Ultrasound guidance in regional anaesthesia is increasingly being used; this has led to approaches to the sciatic nerve block, femoral nerve block (FNB), adductor canal block (ACB), and obturator nerve block being continually reevaluated and increasingly motivated as an element of multimodal analgesia regimen in many operations, including knee arthroscopy.^5,6^

Traditionally FNB has been used as a gold standard for preoperative analgesia following knee surgery. In the last decade, the ACB has been increasingly performed as an alternative technique to FNB with achieving similar pain relief, and the use of FNB was overshadowed by ACB. In addition, ACB has the advantage of early functional recovery with minimal effect on quadriceps muscle strength compared to the FNB.^7^ Although there is a common consensus on effectiveness of ACB, the optimal location of ACB placement is still controversial. There have been multiple trials that have suggested an important role for proximal, mid, and distal ACB techniques in pain control for patients undergoing knee surgery, with both optimism and concerns among physicians.^8,9^

In this present study, we aimed to demonstrate the efficacy of proximal, mid, and distal ACB blocks in elective knee arthroscopy. The primary outcome of this study was to compare tramadol consumption at 24 hours of post-operative period. Secondary outcomes were to assess pain intensity, nausea and vomiting, and patient satisfaction in the post-operative period.

## Methods

### Study Design

This study was approved by the Ethics Committee of the Dışkapı Yıldırım Beyazıt Training and Research Hospital (108/03- 05.04.2021). We included the patients between April 2021 and June 2021. All patients provided written informed consent for study procedures and future data publication. This study included 90 patients, American Society of Anaesthesiologists classification I-III, aged between 18 and 80 years who were undergoing elective arthroscopic knee surgery. Patients with the recent use of opioids due to chronic pain therapy, neuropathic or chronic pain, allergies to local anaesthetics, unwillingness to participate, or who were unable to give consent were excluded from the study.

**Figure 1.**
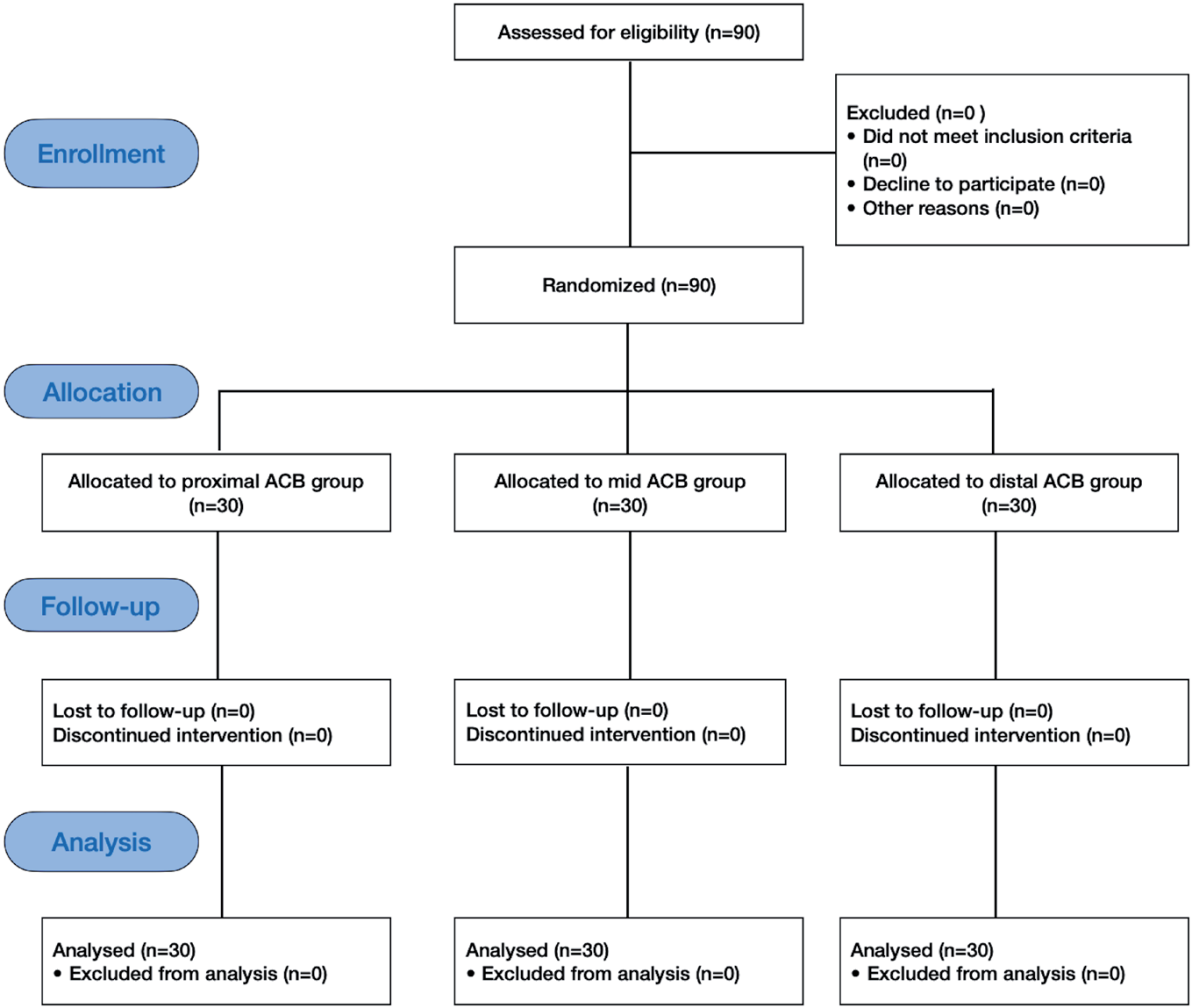
Flow chart of the study.

The flowchart of this trial is shown in Figure 1.

### Study Groups

Using a 1 : 1 : 1 ratio with a computer-generated randomisation concealed in sealed envelopes, each patient was randomised into 1 of the 3 treatment groups: proximal, mid, or distal ACB. Randomisation assignments remained concealed until block performance. Except for the anaesthesiologist (AT) who performed the blocks, all other researchers, nurses providing pre-operative care, and enrolled patients were kept blinded to randomisation.

### General Anaesthesia Management

All patients received a standardised general anaesthesia procedure. In the operating room, IV lines and standardised monitoring comprising SpO2, continuous electrocardiography, and non-invasive blood pressure were applied. Intravenous propofol 2-3 mg kg^−1^, fentanyl 1 μg kg^−1^, and rocuronium bromide 0.3 mg kg^−1^ were used for general anaesthesia induction. A laryngeal mask airway was used in providing the airway management. Anaesthesia maintenance was provided with remifentanil infusion and sevoflurane 2% in a 50 : 50 mixture of oxygen and air. All block applications were performed after general anaesthesia in sterile conditions. In order to prevent post-operative nausea and vomiting, granisetron 1 mg was administered intraoperatively. Nausea and vomiting were recorded as present/no during the first 24 hours after surgery.

### Adductor Canal Block Technique

Adductor canal block was performed on the affected side in supine position by the same AT. A high-frequency linear array ultrasound (Sonosite M-Turbo) transducer was first placed on the inguinal fold. By tilting the probe, the femoral artery was detected in the short axis and the probe was advanced distally from the apex of the femoral triangle. The proximal block position was designated at the site as the intersection of the medial borders of the sartorius muscle and the medial border of the adductor longus muscle by ultrasound image. The distal block position was determined by sliding the ultrasound probe distally where the femoral artery moved away from the sartorius muscle and advanced deep into the adductor hiatus. Mid-level block location was determined as the place where the femoral artery was parallel to the medial border of sartorius muscle, in the middle of the proximal and distal blocks. A 21-gauge 85-mm Stimuplex needle (Stimuplex A50; B. Braun, Germany) was advanced from the lateral to medial direction using an in-plane ultrasound technique. After negative aspiration of blood, 20 mL of bupivacaine (0.375%) was administered to the adductor canal.

### Standard *** Analgesia Protocol***

All patients received standard intraoperative and post-operative analgesia protocols. Dexketoprofen 50 mg IV was administered intraoperatively, before the end of surgery. After transfer to the recovery room, a patient control analgesia device which infuses tramadol 3 mg mL^−1^ with no basal infusion, 20 mg bolus dose of tramadol, and a 20 min lock-out time was provided to all patients. Visual analogue scale (VAS) was used to assess the severity of post-operative pain. IV dexketoprofen 50 mg was planned for breakthrough pain if VAS ≥ 4.

## Outcome Measurements

This present trial primarily aims to assess the analgesic requirements including opioid consumption and rescue analgesia in the post-operative 24 hours. Secondary outcomes include the mean changes in pain scores at rest and during activity, motor block of lower limb, quality of sleep (bad/moderate/good), and patient satisfaction.

A 10-mm VAS, where 0 mm indicated no pain and 10 mm indicated severe pain, is a widely used scale to assess the severity of pain felt by a patient. Visual analog scale pain score was recorded upon postanaesthesia care unit (PACU) admission and at 2nd, 4th, 8th, 12th, and 24th hours.

The Bromage score at PACU, 2, 4, 8, 12, and 24 hours post-operatively was evaluated as follows: no motor nerve block = 0 point; able to move feet and knee = 1 point; able to move feet only = 2 points; and inability to move hip, knee or feet = 3 points.

Patient satisfaction with analgesia was recorded using a 5-point Likert scale (0, not at all satisfied; 5, very satisfied). Quality of sleep at post-operative period was asked as a verbal assessment. We recorded the response as “poor,” “moderate,” and “good.” Nausea and vomiting were questioned at the post-operative recovery room. Nausea was recorded as present/no vomiting during the post-operative 24 hours.

### Sample Size and Statistical Analysis

Sample size calculation was performed using G*Power software version 3.1.9.7 (Heinrich-Heine-Universität, Düsseldorf, Germany) according to our preliminary study data. At our clinic, we conducted a preliminary study of 10 patients who showed a mean (± SD) tramadol consumption of 95.4 mg (±26.4) at the 24th hour post-operatively. Using our preliminary results and considering the tramadol consumption as the primary outcome, a sample size of 25 patients in each group was determined to be necessary in order to detect a 20% between-group difference in post-operative tramadol requirements at 24 hours, and a level of 0.05, and power of 80%. Considering a 20% dropout probability, we included 30 patients in each group. Data analyses were performed using Statistical Package for Social Sciences software version 25.0 (IBM SPSS Corp., Armonk, NY, USA). Shapiro–Wilk test was performed to evaluate whether variables conformed to a normal distribution. Normally distributed quantitative demographic data were expressed as the means ± standard deviations (SD), and non-normally distributed data were expressed as medians (interquartile ranges). Categorical data were presented as counts (percentages). Comparison of independent groups for nonparametric data was performed with Kruskal–Wallis test and Bonferroni correction was applied to adjust the *P* values. Homogeneity of variances was evaluated with Levene’s test. Analysis of variance tests were performed to compare the mean changes in outcome measurements, and the post hoc Tukey and Tamhane tests were performed to evaluate where the difference between the block groups originated from. Statistically, significance was accepted as *P*-value <.05.

## Results

In this present study, 90 patients were evaluated and completed their follow-up. The patients’ baseline demographic data and clinical characteristics were similar in both groups (Table 1). All surgeries were performed uneventfully. No block-related adverse events such as soreness or hematoma, incidents of falls, or local anaesthetic systemic toxicity were reported in any of the patients.

**Figure 2.**
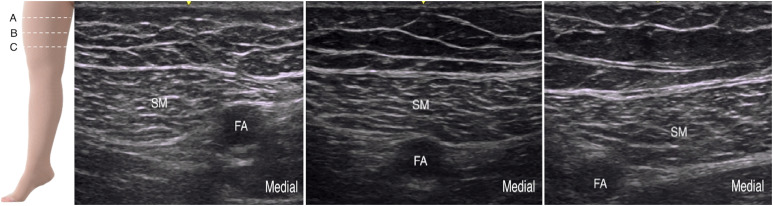
Relative ultrasound probe positions and sonographic configurations of the (A) proximal ACB, (B) mid-ACB, (C) distal ACB. ACB, adductor canal block; FA, femoral artery; SM, sartorius muscle.

Mean tramadol consumption in proximal ACB, mid-ACB, and distal ACB was 98.7 mg (83.6-113.7), 175.3 mg (156.2-194.4), and 222 mg (196.4-247.6), respectively (Table 2) (Figure 2). Proximal ACB group significantly reduced opioid consumption compared to the mid-ACB group (*P* < .001), and mid-ACB group provided significantly decreased opioid consumption than the distal ACB group (*P* = .004).

Analyses between adductor canal block levels showed that there was a significant difference in terms of rescue analgesic requirements during the follow-up period (*P *< .05) (Table 2). The need for rescue analgesic drug was detected in 10 patients in the distal ACB group, while 4 patients in the mid-ACB group and 1 patient in the proximal ACB group (*P* < .05).

When the mean VAS scores at rest were analysed, a significant difference was found between all groups at the 0th, 2nd, 4th, 8th, 12th, and 24th hour (*P* < .001). Post hoc analyses demonstrated that the mean VAS score in the distal group was higher when compared to the mid and proximal groups at each follow-up point (*P* <.001). The VAS scores were significantly lower in the proximal ACB group compared to patients in the mid-ACB group at each follow-up interval; however, there was no difference at the 24th hour (Table 3).

The Bromage score was 0 at 0th, 2nd, 4th, 8th, 12th, and 24th hour in all groups. Post-operative nausea was recorded in 3 patients only in the distal ACB group during 24 hours (*P* < .05). The quality of sleep was similar between groups (Table 2). Finally, analyses demonstrated a significant difference between the groups for patient satisfaction, and this favoured the proximal ACB group (*P* < .001) (Table 2).

## Discussion

This randomised controlled trial was designed to evaluate the effectiveness of ACB performed at proximal, mid, and distal locations in arthroscopic knee surgery. Our study findings determined that ACB performed at a proximal location provides significantly better analgesic efficacy and reduced opioid consumption compared to the mid and distal groups without compromising quadriceps motor block.

Multimodal analgesia is a technique that reduces the side effects associated with opioid use and provides quality and adequate analgesia as a result of additive or synergistic effects with the combination of different analgesics.^10^ What clearly attracts the interest of clinicians is the reason for the use of nerve blocks and their effects on the results of therapy in perioperative analgesia. Femoral nerve block and femoral triangle block can provide effective per-operative analgesia in arthroscopic knee surgery.^11,12^ However, the use of femoral nerve blockade has been gradually abandoned related to the increased length of hospital stay and risk of falls following knee surgery in the last decade. Recently, distal locations such as ACB have been used by many ATs exploring a more distal approach to block peripheral nerves that could provide effective pain relief without compromising motor strength.^7,13-15^ Supportively, previous trials evaluating the efficacy of saphenous nerve block reported that saphenous nerve block significantly reduced VAS pain scores at rest and during movement, and total opioid consumption within the first post-operative 24 hours compared with placebo group.^16-18^ Therefore, our goal is to compare the treatment outcomes of ACBs performed at different levels including proximal, mid, and distal levels.

Although there are studies comparing ACB at proximal and distal locations in the literature, there are few studies comparing proximal, mid, and distal locations. In a meta-analysis involving 348 patients, Zhang et al^19^ compared proximal versus distal ACB techniques, and it was found that total opioid consumption, mean VAS pain scores, and block success rate were similar between the block groups. Similarly previous studies that have evaluated different injection locations of ACB for total knee arthroplasty have not shown any differences in postoperative pain scores and cumulative analgesic consumption within the first 24 hours.^20-22^

On the other hand, in a study in the setting of unilateral total knee arthroplasty, Fei et al^23^ placed a catheter using the proximal and the middle targets for the adductor canal block, and they reported significantly lower opioid consumption in the proximal ACB group compared to the mid-ACB group. Recently, in a randomised study performed by Abdallah et al.^9^ ACB technique was applied from the proximal, mid, and distal levels and they reported that the proximal location for ACB provided greater opioid-sparing effects in patients who had undergone knee surgery, with sparing quadriceps motor strength, compared to distal, and middle ACB injection locations. When pain severity at rest was examined using the area under the curve over the first 24 hours, they found statistically significant differences between the 3 groups, the proximal ACB group was associated with the least pain scores followed by the mid-ACB and then the distal ACB groups.^9^ Similarly, we found that ultrasound-guided proximal ACB provided significantly adequate analgesia and decreased tramadol consumption compared to the mid and distal groups following knee arthroscopy.

Enhanced recovery programmes are effective in reducing opioid burden throughout all stages of surgery, thereby minimising adverse effects of opioids such as nausea and vomiting. Regional techniques in the pre- and post-operative period are an integral part of the multimodal pain management protocols and play an important role in reducing exposure to opioids. This present study showed that post-operative nausea incidence was higher in the distal ACB group compared to the others groups, which could be explained by increased tramadol consumption in the distal ACB group. Performing regional block techniques for post-operative analgesia results in reduced opioid requirement and opioid-related side effects as recommended.

Recently, there have been several publications suggesting a role for femoral and adductor canal blocks in providing preoperative analgesic benefits, with each approach having benefits and pitfalls.^8,9,19,20^ Injection level of the block is one of the most important factors influencing local anaesthetic distribution, and cadaver studies were performed to address this question at the ACB. Previous cadaveric studies have shown staining of the saphenous nerve (SN) and genicular branch of the obturator nerve (GBON) using distal ACB.^24,25^ In a recent anatomic cadaver study, Tran et al^26^ administered 10 mL of dye into the proximal adductor canal under USG guidance in seven samples. They found that 10 mL dye injection spared the anterior branches of the vastus medialis nerve which may explain the motor-sparing advantages of the ACB block by maintaining greater vastus medialis muscle function. Additionally, the authors reported staining of the sensory nerves to the knee joint, the posteromedial branch of the nerve to vastus medialis (NVM), the superomedial genicular nerves (SMGN), in addition to SN and GBON. This may explain the fact that ACB at more proximal injection locations provides better analgesia than a distal injection by targeting additional nerves including posteromedial branch of NVM and SMGN supplying the knee joint, while also sparing quadriceps muscle strength. Although Timed Up and Go tests have commonly been used in similar studies to measure the strength of quadriceps muscle, we used Bromage motor block scale which is an observation index mainly used to evaluate the degree of overall motor blockade.^27,28^ The Bromage score was 0 in all groups at each follow-up point. This finding demonstrates that ACB provides sensory block, without motor block measured by Bromage score. As such, we suggest that proximal ACB block, as an easier and safer regional anaesthesia technique, could be a preferable treatment choice for patients undergoing knee surgery to reduce the aforementioned risks.

The current study has some limitations. First, since we followed the patients for the first 24 hours post-operatively, we could not evaluate the treatment outcomes of techniques in the long-term period. Second, although quadriceps muscle strength was commonly assessed with a hand-held dynamometer or Timed Up and Go test in previous studies, we used Bromage motor block scale to evaluate the degree of overall motor block.^29^ Third, in this present study, we used 20 mL of 0.375% bupivacaine, a slightly higher concentration and volume, for ACB. In previous studies performing ACB, bupivacaine has been used with concentrations ranging between 0.166% and 0.5% as a single injection.^6,30-32^ High concentrations or volumes of local anaesthetic may not only increase the duration of block but also increase the risk of motor block by spreading to one of the motor branches of the femoral nerve innervating the quadriceps muscle. We used 0.375% bupivacaine to make it more prominent, if there are differences including duration of the block and motor block in proximal, mid-, and distal ACB. However, motor block was not observed in any study group. Further studies are warranted to compensate for these limitations.

## Conclusion

In this study, our results showed that ACB performed for knee arthroscopy, especially with the proximal adductor canal injection location, decreased opioid consumptions, and better post-operative pain scores are provided. In addition, our study found that ACB involves no post-operative motor blockade as measured by Bromage motor block scale. We also concluded that the proximal adductor canal injection location for ACB is more advantageous in terms of post-operative nausea/vomiting and patient satisfaction.

## Figures and Tables

**Table 1.** Evaluation of the Descriptive Characteristics According to the Groups

Parameters	Proximal ACB (*n* = 30)	Mid ACB (*n* = 30)	Distal ACB (n = 30)	*P*
Female/male (*n*)	7/23	6/24	12/18	.18
Age (year)	37.4 (33.9-41)	35 (31.1-38.8)	38.3 (34.1-42.5)	.45
BMI (kg/cm^2^)	27.1 (26-28.3)	27.5 (25.9-29.1)	27 (25.6-28.5)	.45
Duration of Surgery (minutes)	71.5 (65.7-77.4)	71 (63.5-78.5)	73.5 (65.7-81.2)	.94
ASA I/II (n)	15/15	15/15	10/20	.32

The values are expressed as the means [95% CI] or as absolute numbers. *P* < .05 is considered statistically significant.

ACB, adductor canal block; ASA, American Society of Anaesthesiologists; BMI, body mass index.

**Table 2.** Results

	Proximal ACB (*n* = 30)	Mid-ACB (*n *= 30)	Distal ACB (*n *= 30)	*P^a^*
Tramadol consumption (mg)	98.7 (83.6-113.7)^b,d^	175.3 (156.2-194.4)^c^	222 (196.4-247.6)	**<.001** ^*^
Patients requiring rescue analgesic (*n*)	1/29 ^d^	4/26	10/20	**.006** ^*^
Motor block (yes/no) (*n*)	0/30	0/30	0/30	N/A
Post-operative nausea and vomiting (yes/no)	0/30^d^	0/30^c^	3/27	**.045** ^*^
Satisfaction, Likert (4/5)	0/30^d^	2/28^c^	10/20	***<.001*** ^*^
Quality of sleep (moderate/good)	2/28	2/28	6/24	.165

Data are expressed as the means [95% CI], median [interquartile range] or absolute numbers. Note that Bonferroni adjustment was done. ACB, adductor canal block; N/A, not applicable

*P* values italicised and written in bold represent statistical significance. ^*^ *P* < .05.

^a^Kruskal-Wallis test was performed. ^b^Pairwise comparison of the proximal ACB vs. mid ACB (adjusted* P* < .05). ^c^Pairwise comparison of the mid-ACB vs. distal ACB (adjusted *P* < .05). ^d^Pairwise comparison of the proximal ACB vs. distal ACB (adjusted *P* < .05).

**Table 3.** Evaluation of the VAS Scores According to Groups and Time

	Proximal ACB (n = 30)	Mid ACB (n = 30)	Distal ACB (n = 30)	Overall Group Effect^*^	Mid vs. Proximal	Distal vs. Mid	Distal vs. Proximal
VAS scores at rest, cm				*P*			
0 h	0.37 (0.14-0.60)	1.37 (1.08-1.65)	2.10 (1.69-2.51)	<.001*	.001*	.012*	<.001*
2 h	1.10 (0.83-1.37)	1.90 (1.70-2.10)	2.57 (2.25-2.89)	<.001*	<.001*	.02*	<.001*
4 h	1.37 (1.16-1.57)	2.33 (2.07-2.60)	2.77 (2.48-3.06)	<.001*	<.001*	.043*	<.001*
8 h	1.57 (1.38-1.75)	2.30 (2.04-2.56)	2.90 (2.60-3.20)	<.001*	<.001*	.003*	<.001*
12 h	1.47 (1.25-1.68)	2.00 (1.78-2.22)	2.57 (2.26-2.87)	<.001*	.007*	.004*	<.001*
24 h	1.27 (1.10-1.43)	1.53 (1.32-1.75)	2.00 (1.76-2.24)	<.001*	.165	.005*	<.001*
VAS scores during movement, cm				*P*			
0 h	1.40 (1.04-1.76)	2.53 (2.14-2.92)	3.00 (2.49-3.51)	<.001*	.001*	.256	<.001*
2 h	2.27 (1.97-2.56)	3.20 (2.85-3.55)	3.93 (3.58-4.29)	<.001*	<.001*	.05*	<.001*
4 h	2.50 (2.26-2.74)	3.37 (3.06-3.67)	4.03 (3.78-4.28)	<.001*	<.001*	.001*	<.001*
8 h	2.63 (2.37-2.90)	3.10 (2.83-3.37)	3.87 (3.58-4.16)	<.001*	.042*	<.001*	<.001*
12 h	2.23 (2.05-2.42)	2.97 (2.70-3.23)	3.23 (2.84-3.62)	<.001*	<.001*	.584	<.001*
24 h	1.83 (1.64-2.03)	2.30 (2.02-2.58)	2.63 (2.30-2.97)	<.001*	.043*	.193	<.001*

Data are expressed as median (percentiles 25-75). *P*-values italicised and written in bold represent statistical significance. ^*^ *P* < .05. VAS, visual analogue scale.
